# Mesenchymal Stromal/Stem Cells and Their Extracellular Vesicles Application in Acute and Chronic Inflammatory Liver Diseases: Emphasizing on the Anti-Fibrotic and Immunomodulatory Mechanisms

**DOI:** 10.3389/fimmu.2022.865888

**Published:** 2022-04-07

**Authors:** Ali Hazrati, Kosar Malekpour, Sara Soudi, Seyed Mahmoud Hashemi

**Affiliations:** ^1^ Department of Immunology, Faculty of Medical Sciences, Tarbiat Modares University, Tehran, Iran; ^2^ Department of Immunology, School of Medicine, Iran University of Medical Sciences, Tehran, Iran; ^3^ Department of Immunology, School of Medicine, Shahid Beheshti University of Medical Sciences, Tehran, Iran

**Keywords:** MSCs, exosomes, liver disease, immunomodulation, inflammation, regeneration

## Abstract

Various factors, including viral and bacterial infections, autoimmune responses, diabetes, drugs, alcohol abuse, and fat deposition, can damage liver tissue and impair its function. These factors affect the liver tissue and lead to acute and chronic liver damage, and if left untreated, can eventually lead to cirrhosis, fibrosis, and liver carcinoma. The main treatment for these disorders is liver transplantation. Still, given the few tissue donors, problems with tissue rejection, immunosuppression caused by medications taken while receiving tissue, and the high cost of transplantation, liver transplantation have been limited. Therefore, finding alternative treatments that do not have the mentioned problems is significant. Cell therapy is one of the treatments that has received a lot of attention today. Hepatocytes and mesenchymal stromal/stem cells (MSCs) are used in many patients to treat liver-related diseases. In the meantime, the use of mesenchymal stem cells has been studied more than other cells due to their favourable characteristics and has reduced the need for liver transplantation. These cells increase the regeneration and repair of liver tissue through various mechanisms, including migration to the site of liver injury, differentiation into liver cells, production of extracellular vesicles (EVs), secretion of various growth factors, and regulation of the immune system. Notably, cell therapy is not entirely excellent and has problems such as cell rejection, undesirable differentiation, accumulation in unwanted locations, and potential tumorigenesis. Therefore, the application of MSCs derived EVs, including exosomes, can help treat liver disease and prevent its progression. Exosomes can prevent apoptosis and induce proliferation by transferring different cargos to the target cell. In addition, these vesicles have been shown to transport hepatocyte growth factor (HGF) and can promote the hepatocytes’(one of the most important cells in the liver parenchyma) growths.

## 1 Introduction

Inflammation is an immunological condition that has an important role in controlling microbial infections and is the basis of many natural physiological events of the body in healthy conditions (1). This physiological process plays a vital role in embryo implantation, tissue repair, fetal growth, etc. (2). These inflammations must be carefully regulated to maintain the homeostasis of various tissues and organs. Failure to control inflammation can lead to multiple injuries to the tissues involved and lead to the loss of part or all of that tissue (3). Hemostatic inflammation is an integral part of liver health, and treatment strategies should focus on reversing pathological inflammation to hemostatic inflammation (4).

**Figure 1:**
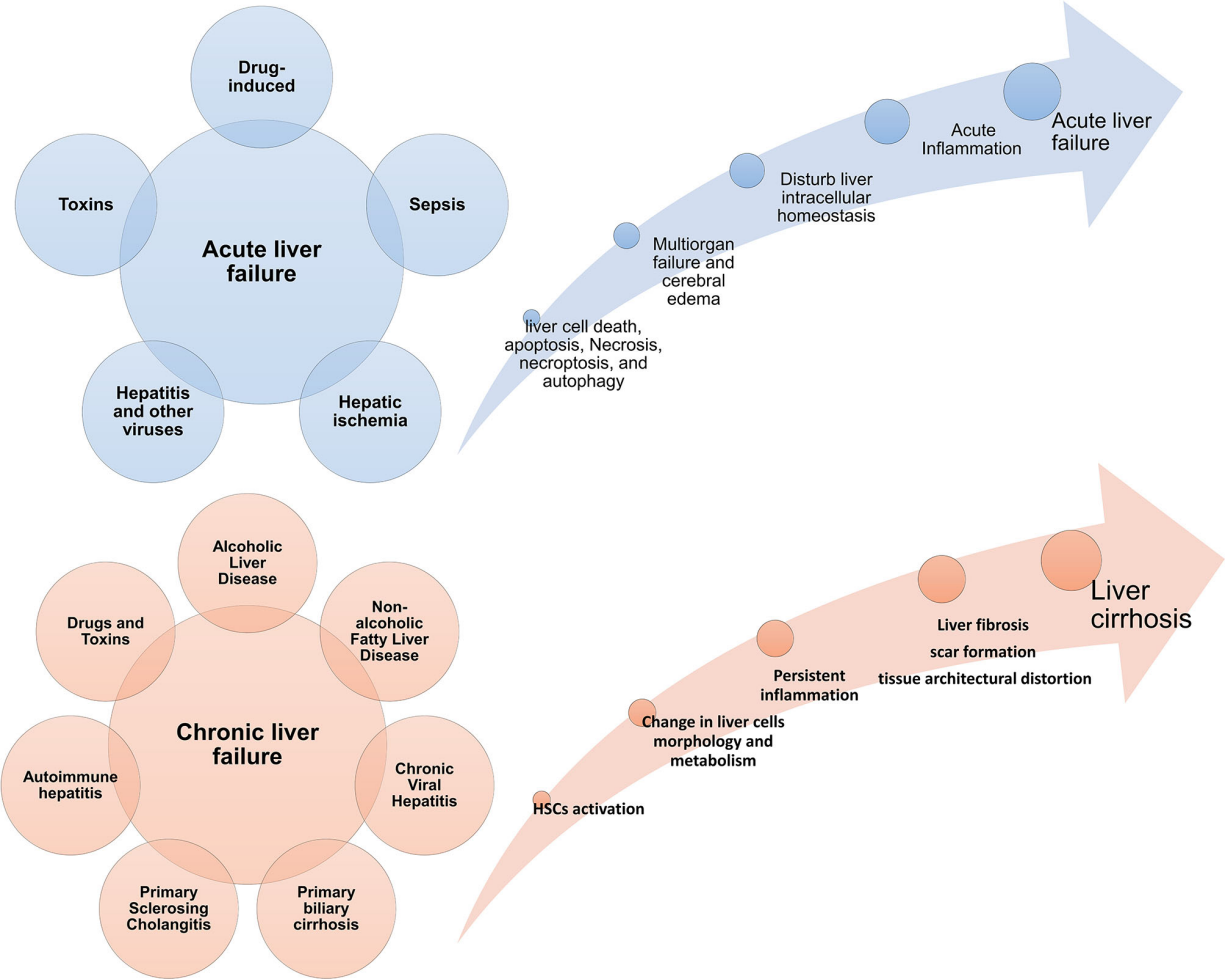
Acute and chronic liver diseases. Diseases Different conditions can cause acute and chronic inflammatory conditions in the liver. If the damages are not controlled in the acute stage, they progress to a chronic state and are associated with fibrosis and cirrhosis of the liver tissue. Failure in acute liver disease treatment can also lead to the onset and progression of liver cancer; HSC, hepatic stellate cell.

The liver is one of the vital organs that has a great ability to regenerate itself due to liver cells’ characteristics (5, 6). But when the amount of damage is so much and exceeds liver cells’ ability to regenerate, the function of this tissue is impaired (7). Acute liver failure (ALF) is more common in adolescents and is associated with high mortality (8). In ALF, the metabolic and immunological function of the liver is impaired. It presents with manifestations such as blood coagulation defects, cardiovascular instability, liver encephalopathy, susceptibility to infection, and progressive multiple organ failure (9, 10). The interval between the onset of symptoms and the onset of hepatic encephalopathy distinguishes different forms of the disease (11). In this way, if the time between them is a few hours, it is called hyperacute liver failure (12), but if this interval occurs more slowly and lasts from a few days to a few weeks, it is called acute or sub-acute damage (13). Fibrosis is an intrinsic response to injury that maintains the integrity of the various organs involved in tissue damage due to extensive necrosis or apoptosis (14). Chronic liver disease occurs during persistent inflammation and is associated with the destruction and regeneration of the liver parenchyma, which eventually leads to fibrosis and cirrhosis. There are many different causes for chronic liver disease, autoimmune diseases, including long-term alcohol abuse, toxins, infections, genetic and metabolic disorders (15). For example, during myocardial infarction, fibrosis and collagen fibres become apparent by the end of the first week, preventing heart rupture by maintaining the heart’s structure (16). Tissue fibrosis is also seen in the kidney after ischemia (17) and in the lung after H5N1 influenza and COVID-19 infection (18–20). **Figure 1** summarizes some acute and chronic liver diseases and the main mechanisms involved in these diseases development (**Figure 1**). It is worth noting that various treatments can prevent the progression of liver fibrosis (21) but do not lead to the reversion of fibrous tissue, and there is no definitive treatment for fibrosis.

There is currently no specific drug for treating liver disease, and many of the current treatments are used only in very acute cases due to their side effects (22). Therefore, there is an urgent need to find appropriate treatment methods to prevent liver disease progression and repair liver tissue. Today cell therapy is one of the fields that has raised many hopes and interests in the field of regenerative medicine. Cells such as mesenchymal stromal/stem cells, hepatocytes, hematopoietic cells, immune system cells and endothelial progenitor cells have been used in various studies to treat liver disease (23).

There are advantages and disadvantages to using each of these cells, which are summarized in **Table 1**. Among these cells, mesenchymal stem cells have been used extensively in studies due to their desirable properties and have shown promising results. The desirable characteristics of these cells are various isolation sources, easy isolation methods, easy culture methods, expansion and storage, low immunogenicity, self-renewal ability, ability to differentiate into different cell types, immunomodulatory properties, The ability to produce soluble factors such as exosomes and growth factors and the ability to migrate to the site of tissue damage were noted (39, 40). Due to the favourable properties of MSCs-derived extracellular vesicles (EVs), the therapeutic ability of these vesicles has been investigated. This review discusses the effect of inflammation on the progression of liver disease, the role of liver cells and immune cells in the pathogenesis of the liver disease, and studies of MSCs and their EVs (mechanisms).

**Table 1:** Advantages and disadvantages of each cell in therapeutic applications.

Cell type	Advantage	Disadvantage	Ref.
Endothelial progenitor cells (EPCs)	Anti-fibrotic and pro-regenerative properties	Complicated isolation process, unclear clinical efficacy	(24, 25)
Hematopoietic stem cells	Pluripotency Potential to self-renew	Requires bone marrow aspiration Linage derivation (such as derivation to macrophages)	(26, 27)
Hepatoblasts (Fetal liver Stem Cells)	1. these cells are bipotent, being able to give rise to both hepatocytes and bile duct cells	Rarity of hepatoblasts 0.1% of fetal liver mass Presence of oval cells in adult liver (make isolation an expansion difficult)	(28–30)
Hepatocytes	Key metabolic and synthetic cells of the liver Suitable for replacing enzyme deficiency Suitable for replacing metabolic disorders	Donor shortages Limited engraftment and proliferation Infection risk	(31, 32)
Immune cells	Relatively easy to isolate and culture	Potential ability to induce inflammatory storms	(33)
Induced pluripotent stem cells (iPS)	an unlimited source to produce hepatocytes-like cells *In vitro* Lack of potential issues of allogenic rejection	Ethical concern Malignancy potential Low production efficiency	(34, 35)
MSCs	Relatively easy to isolate and culture pluripotency immunomodulatory and anti-inflammatory properties Anti-fibrotic function Extracellular signaling Allograft potential Diffreniatonal ability	Pro-tumor potential Risks of isolation procedures Malignancy potential Risk of undesired migration to other organs such as lung and kidney	(36–38)

## 2 Inflammation Role in Liver Diseases (LDs)

Under physiological conditions, the liver constantly contacts food-derived foreign proteins, drugs, chemicals, toxins, and intestinal microbiota (41). Kupffer cells (KCs) and dendritic cells (DCs), as well as circulating immune cells such as bone marrow-derived macrophages, natural killer (NK) cells, and neutrophils (42), play an important role in the formation of the liver immune microenvironment and hepatic immune responses (43).

Kupffer cells become highly active in the liver during this disease and produce inflammatory cytokines, including IL-1, IL-6, and TNF-α. In addition, due to the activation of other immune system cells that are recruited from the bloodstream to the liver tissue, systemic inflammation develops that can stimulate necrosis and apoptosis in hepatocytes (44, 45). Overall, inflammation, immune cells and liver cells inflammatory responses play an essential role in developing liver-related diseases (46).

Hepatic steatosis, or fatty liver disease (FLD), is the most common liver disease in the United States (47), occurs in response to alcohol, chemotherapy, toxins such as vanilla chloride, and insulin-related metabolic syndrome and can lead to liver tissue damage (48). This damage is associated with inflammation and fibrosis, alters hepatocyte gene expression, and leads to increased TLRs ligands, TGF-β, CXCL10, and IL-1A receptors (49). In hepatitis, activation of the NF-κB signaling pathway in activated hepatocytes has also been observed. In this pathway, the activated cells release several pro-inflammatory cytokines and chemokines such as TNF-α, IL-6, and CCL2, which mediate liver inflammation (50). Inflammation can lead to necrosis and necroptosis of liver cells (especially in hepatocytes) and result in releasing of a group of molecules called alarmin from them. Alarmins such as the high-mobility group B1 (HMGB1), IL-33, ATP, and formyl peptide is released from necrotic cells (51). In fact, ATP and formyl peptide act as absorbers for neutrophils to the site of tissue damage in the liver. Activation of inflammasomes by reactive oxygen species (ROS) from damaged cells and other damage-associated molecular patterns (DAMPs) can also play a role in liver inflammation (52, 53). As a result of liver inflammation, extensive necrosis and apoptosis in hepatocytes eventually lead to loss of liver function (54). Therefore, controlling inflammatory responses is of particular importance and, if left untreated, can lead to fibrosis (55).

Fibrosis that has occurred in chronic liver disease and inflammation has been extensively studied, but its underlying mechanism in acute liver failure is still unclear (56). The progression of fibrosis leads to cirrhosis, hepatocellular carcinoma, liver failure, and portal hypertension (57). Liver fibrosis is caused by the activation of hepatic stellate cells (HSCs) and the extracellular matrix (ECM) deposition (58). When these cells are activated, the amount of vitamin A and adipogenic-related transcription factors decreases and leads to their differentiation into myofibroblasts, which are the main source of ECM production in liver fibrosis (59). In addition, HSCs contribute to the progression of liver fibrosis by producing inflammatory cytokines and chemokines (60). Immune system cells, especially KCs and circulating macrophages, play a significant role in the TGF-β1-mediated activation of the liver HSCs and increase their survival by the NF-κB-dependent manner (61). reactive oxygen species (ROS) production by KCs and macrophages is another fibrosis stimulant in hepatitis that stimulates the production of collagen 1 in HSCs/myofibroblasts (62).

In response to lipopolysaccharide (LPS), HSCs express chemokines such as IP-10, MCP-1, MIP-1α, MIP-1β, MIP-2, RANTES, and adhesive molecules E-selectin, ICAM-1, VCAM-1, and leading to migration of immune system cells into the liver (63). If liver fibrosis is left untreated, Cirrhosis develops. Cirrhosis is the end stage of progressive fibrosis that lacks effective and comprehensive medical treatment (64). Liver cancer is another consequence of fibrosis and liver inflammation.

Studies have identified the importance of inflammation-related signaling pathways and transcription factors, including STAT3 (55) and NF-κB (65), in liver cancer progression by four mechanisms. (1) Increases the expression of epithelial-to-mesenchymal transition (EMT) associated genes, including matrix metalloproteinases (MMPs) 2 and 9 (in breast cancer), E-cadherin, TWIST (in nasopharyngeal cancer), and cathepsins B. (2) Stimulates angiogenesis and increases tumor growth by regulating the expression of vascular endothelial growth factor (VEGF) (66). (3) Increases the expression of Bcl-2 members and cFLIP families and helps neoplastic cells proliferation and inhibit their apoptosis (67). (4) Increase production of inflammatory cytokines such as IL-1α, TNF-α, IL-1β, EGF-R, IL-6, and CCL2 (50, 68).

## 3 The Role of Liver Cells in Liver Inflammation

**Figure 2:**
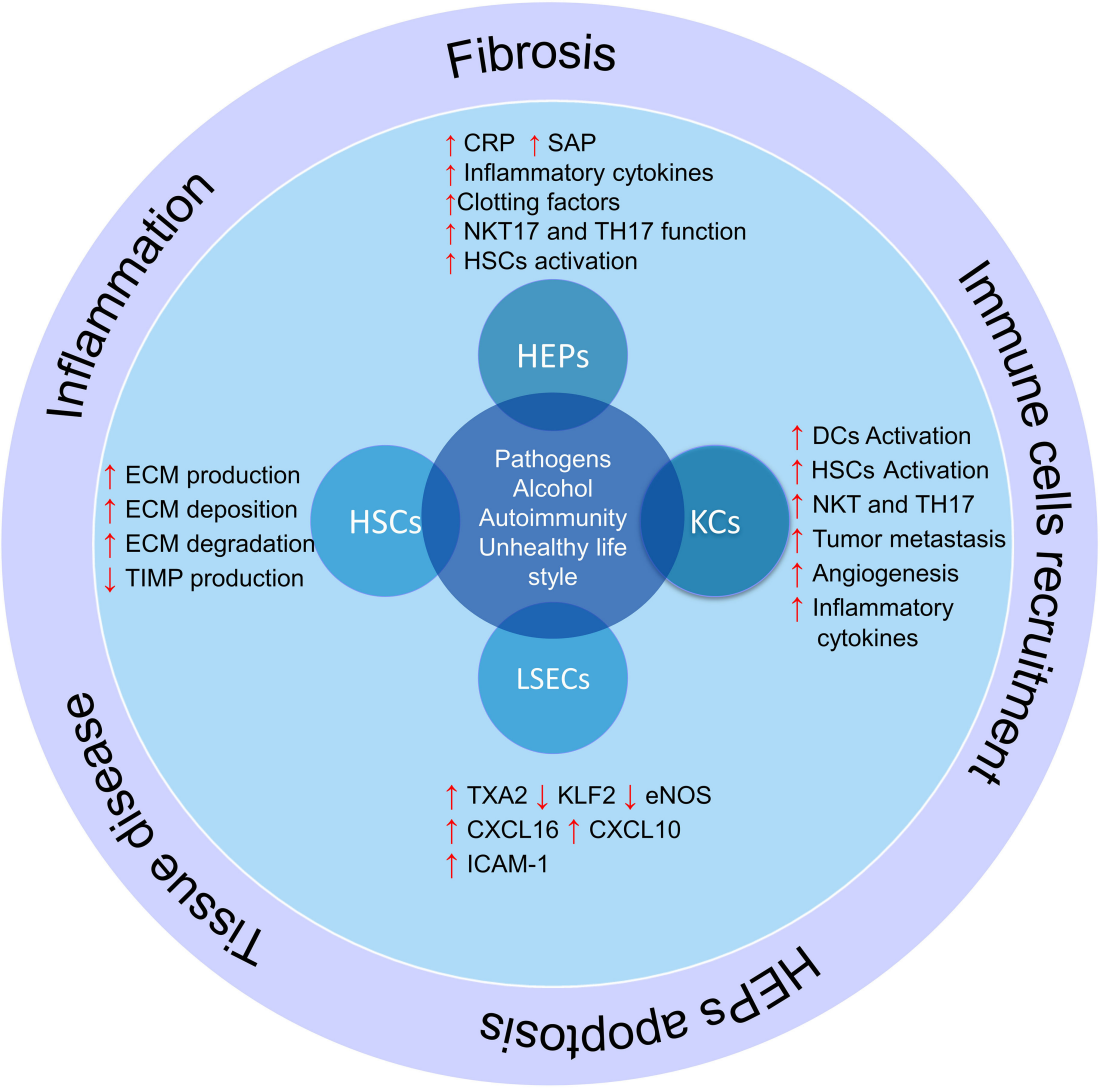
The role of liver cells in inflammation and liver damage. Liver cells help produce and activate immune cells by producing cytokines and inflammatory chemokines. On the other hand, hepatic stellate cells produce fibrosis of the liver tissue and lose their function by producing different components of the extracellular matrix and differentiation into myofibroblasts in inflammatory conditions; HSCs, hepatic stellate cells; KCs, Kupffer cells; LSECs, hepatic sinusoidal endothelial cells; HEPs, Hepatocytes; DCs, Dendritic cells; ECM, extracellular matrix; TXA2, Thromboxane A2; KLF2, Krüppel factor 2 transcription factor; TIMP, Tissue inhibitors of matrix metalloproteinases.

There are some major types of cells in the liver that regulate liver different functions, including hepatocytes, hepatic sinusoidal endothelial cells (LSECs), bile epithelial cells (cholangiocytes), HSCs, and KCs (69). Each of these cells performs their specific function, and a defect in their function leads to a defect in the function of the liver (70). In addition, some of these cells play vital roles in regulating the liver microenvironment and participate in inflammation and immune responses (**Figure 2**).

### 3.1 Hepatocytes

Hepatocytes are the most abundant liver cells, which make up about 90% of its biomass, and are one of the main culprits in the liver’s inflammatory response (71). In pathological conditions, hepatocytes produce different chemokines that trigger the immune system cells and create a complex local reaction at the site of infection (72). Hepatocytes express a large number and variety of pattern recognition receptors and identify different types of molecular patterns with pathogens and damage (PAMP, DAMP) (73). (1) Cell surface receptors such as quasi-tuft receptors such as TLR2,4; (2) Endosomal receptors such as TLR3 and (3) Cytoplasmic receptors, such as stimulators of IFN [STING] genes, members of the nucleotide-binding oligomerization domain (NOD) family, and retinoic acid 1 (RIG-1) induction gene (72). During the acute phase response, circulating levels of several proinflammatory cytokines, including IL-1a, TNF-a, and IL-6, increase (74). The most important cytokine affecting hepatocytes’ function is IL-6 (75) and induces the expression of acute-phase proteins including serum amyloid A, reactive protein C, haptoglobin, α1-antichymotrypsin and fibrinogen (76).

Hepatocytes also interact with innate and acquired immune cells and activate or inhibit their responses by expressing different ligands. These cells express MIC-A, MIC-B, and CD1d (in mice, not humans) and thus interact with NK and NKT cells (76, 77). There is also evidence that these cells interact with T cells to alter their responses and regulate their function (78, 79).

### 3.2 Kpffer Cells (KCs)

Kupffer cells make up 20% of non-parenchymal cells in the liver and are located around the portal vein (80). Therefore, due to the potential and different functions of these cells, they play an important role in liver immunology, tissue homeostasis, as well as various liver diseases, including liver cancer, ischemia-reperfusion (I/R) injury, liver fibrosis and infectious diseases (81). Actually, different signals in the liver microenvironment, such as PAMPs and DAMPs, affect the function of Kupffer cells. For example, HMGB1 released by hepatocytes can activate KCs in Acetaminophen-induced liver injury (AILI), Non-alcoholic steatohepatitis (NASH), and I/R injury (82, 83).In liver diseases, KCs interact with other liver cells such as hepatocytes, cholangiocytes, LSECs, HSCs, and other immune cells. This interaction can exacerbate tissue damage or help to repair and regenerate the liver tissue depending on the type of cells and signalling pathways (81).

### 3.3 Hepatic Stellate Cells

HSCs, as another main cell of liver tissue, make up 13% of sinusoidal cells and 5 to 8% of all liver cells that perform various functions (84). Among the physiological roles of these cells is (1) synthesis of ECM, (2) regulation of sinusoidal blood flow, (3) storage of vitamin A and (4) synthesis of metalloproteinases (85). After various damage to liver tissue (cause inappropriate function) and apoptosis of its cells, HSCs loses their fat-rich granules and differentiate into trans-smooth muscle alpha-actin containing myofibroblasts, producing ECM and inflammatory cytokines (86). **Table 2** shows the different functions of these cells and their role in liver inflammation in various diseases (**Table 2**).

**Table 2:** Hepatic satellite cells role in different liver disease.

Type of disease	Inducing factors	Mechanism	Ref.
HCC	Hepatitis, Alcoholic liver diseases, and NASH	contribute to the formation of tumor microenvironment favorable for tumor growth Activated HSCs in the tumor stroma continuously produce ECM production of soluble factors favoring tumor growth, such as hepatocyte growth factor and TGF-β production of proangiogenic factors such as vascular endothelial growth factor-A (VEGF-A) and MMP9	(87)
I/R liver injury	Ischemia and reperfusion in liver transplantation, imbalances in pH and electrolytes	interaction of CD4^+^ T cells with HSCs before entering the hepatic parenchyma induce the expansion of regulatory T cells (Protective)	(88–90)
Immune-induced hepatitis	Concanavalin A (ConA) and LPS	control CD4^+^ T cell trafficking to liver parenchyma contribution of HSCs to massive production of inflammatory cytokines and chemokines by intra- and extrahepatic immune cells in a paracrine manner	(91–93)
NASH	Increased intestinal permeability	The activation of HSCs by TLR4 (the production of chemokines and the expression of adhesion molecules ICAM-1 and VCAM-1 incensement in the interaction between HSCs and Kupffer cells	(94)
Viral hepatitis	Hepatitis B and C virus	inflammatory and fibrogenic responses by HSCs cell proliferation and nonstructural proteins augment ICAM-1 expression and chemokine production through the NF-κB induction cell migration and activation of several inflammatory pathways in response to CCL21 secreted by activated dendritic cells	(95)

HCC, Hepatocellular carcinoma; ECM, extracellular matrix; HSCs, hepatic stellate cells; MMPs, Matrix metalloproteinase; MSCs, Mesenchymal stromal/stem cells; I/R, Ischemia/reperfusion; NF-kB, Nuclear factor kappa-B; LPS, Lipopolysaccharide; TGF-β, Transforming growth factor-beta; NASH, Non-alcoholic steatohepatitis; TLR, Toll-like receptor.

### 3.4 Liver Sinusoidal Endothelial Cells

LSECs are one of the most specialized endothelial cells with high permeability and have the highest capacity for endocytosis that interface with blood cells on the one side and hepatocytes and HSCs on the other (96). Under pathological conditions, the function of LSECs is changed (97). Capillarization is a process that LSECs acquire vasoconstrictive, thrombotic, and proinflammatory properties (98). During I/R injury and hypoxia due to disruption of blood flow to the liver, LSECs become rounded cells due to vacuolation of the nuclei and reduced ATP supply. In addition, stress on blood flow to the liver leads to a decrease in the Krüppel factor 2 transcription factor (KLF2) in LSECs and its target genes, including the endothelial nitric oxide synthase (eNOS) (99). Since the expression of ICAM-1 and Stabilin-1 in LSECs increases during inflammation, the transmigration of immune cells increases to the liver tissue (100, 101). Expression of mentioned adhesion molecules by LSECs leads to platelet adhesion, vascular microthrombi formation, and platelet-activating factor (PAF) production by platelets, which activates neutrophils and increases ROS production and damages liver tissue (97).

In chronic inflammation, these cells lose their function and play a key role in the onset and progression of chronic liver disease through four processes: sinus capillary formation, angiogenesis, angiocrine signals, and vasoconstriction (102). Decreased NO produced by eNOS by decreased KLF2 activity and increased ROS leads to HSC activation, which is associated with the production and deposition of ECM, increased vasoconstrictors including TXA2 and endothelin-1, and the production of proinflammatory cytokines (103–105).

## 4 Mesenchymal Stem Cells Application in LDs

**Figure 3:**
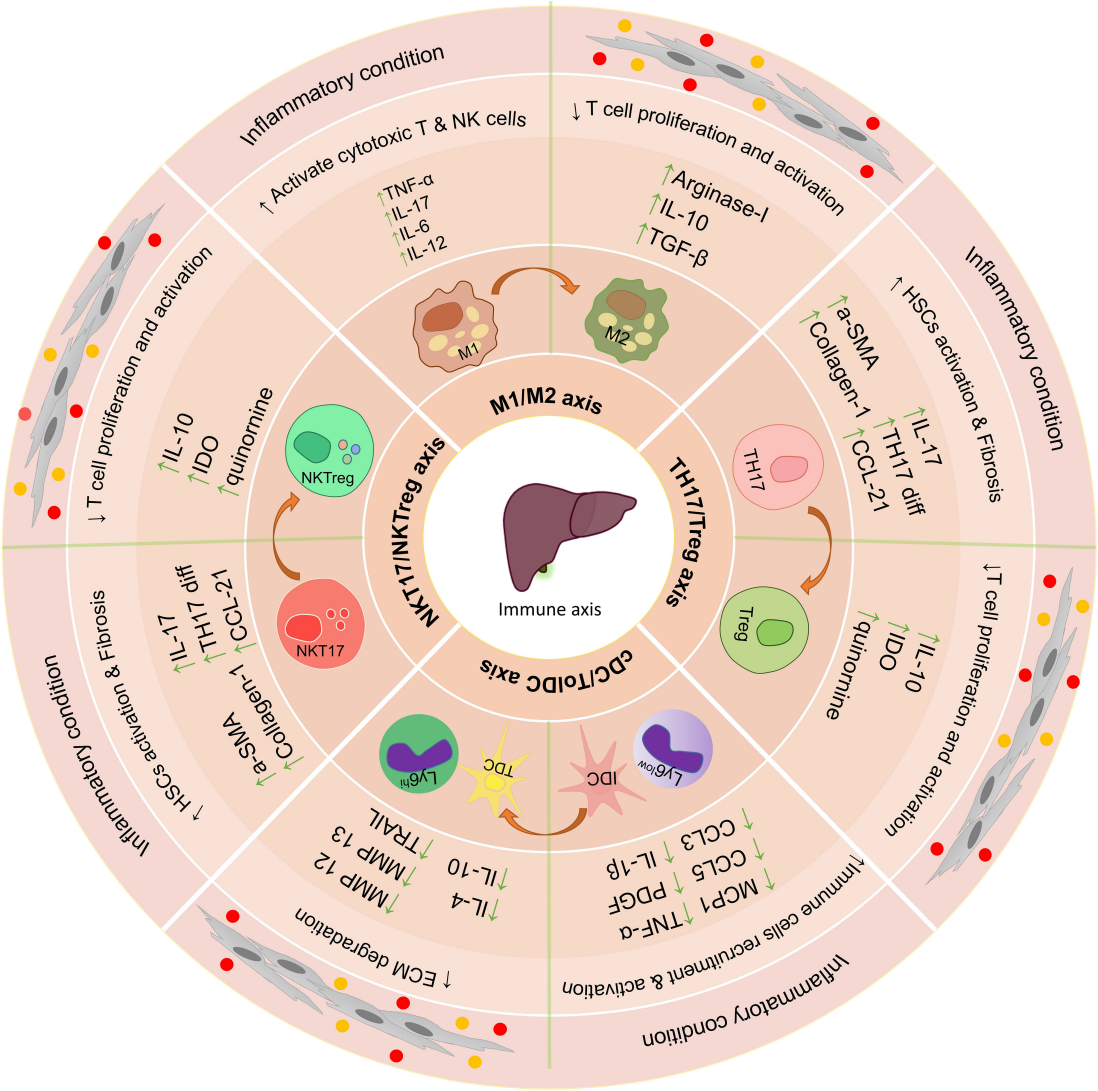
The effect of using mesenchymal stem cells and extracellular vesicles on the important axis of the immune system in the liver. Immunomodulatory properties of mesenchymal stem cells and their extracellular vesicles lead to the suppression of inflammatory responses in the liver’s microenvironment and increase anti-inflammatory responses. Some of the most important axis of the immune system involved in liver disease is TH17/Treg, NKT17/NKTreg, and M1/M2. The use of mesenchymal stem cells and extracellular vesicles leads to suppression of function Th17, NKT17, M1, and IDC and increase the differentiation and function of Treg, NKTreg, M2, and Tol DCs; HSCs, hepatic stellate cells; KCs, Kupffer cells; LSECs, hepatic sinusoidal endothelial cells; HEPs, Hepatocytes; Tol DCs, Tolerogenic dendritic cells; cDC, Classic dendritic cells; ECM, extracellular matrix; MSCs, Mesenchymal stromal/stem cells;, M1 Macrophage type 1; M2, Macrophage type 2; MMPs, Matrix metalloproteinase; EVs, Extracellular vesicles.

*In vitro* studies and co-culture of MSCs with liver tissue-derived cells can increase our understanding of their therapeutic properties and their effectiveness in the treatment of liver disease (106) (**Figure 3**). *In vivo* injections of MSCs into animals to treat various liver diseases have shown very promising results (107). These cells exert their therapeutic functions in laboratory models through various mechanisms (108), and in many studies, only some of the therapeutic aspects of these cells have been addressed. Overall these studies show that MSCs exert their therapeutic effect by modulating the immune environment and increasing the regeneration of liver tissue.

### 4.1 The Role of MSCs in Modulating Immune and Inflammatory Responses

**Figure 4:**
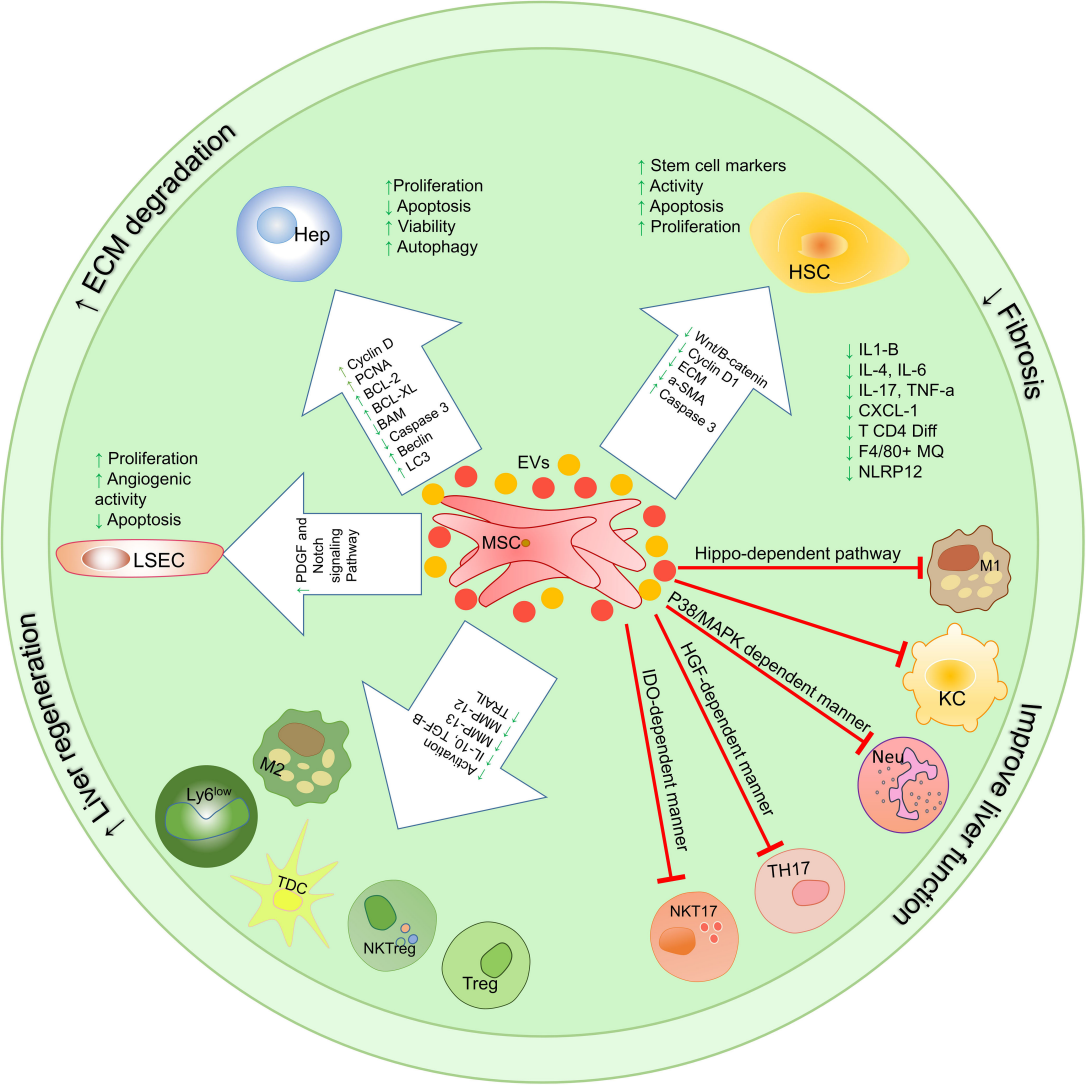
Therapeutic mechanisms involved in using mesenchymal stem cells and their extracellular vesicles on the function of liver cells and immune system cells. MSC-EVs exert their therapeutic functions through 3 mechanisms that have been studied *in vitro*, pre-clinical, and clinical trials. These mechanisms include (1) proliferation induction/apoptosis suppression, (2) modulation of immune system responses, (3) reduction of fibrosis; HSCs, hepatic stellate cells; KCs, Kupffer cells; LSECs, hepatic sinusoidal endothelial cells; HEPs, Hepatocytes; TolDCs, Tolerogenic dendritic cells; cDC, Classic dendritic cells; ECM, extracellular matrix; MSCs, Mesenchymal stromal/stem cells;, M1 Macrophage type 1; M2, Macrophage type 2; MMPs, Matrix metalloproteinase; EVs, Extracellular vesicles.

Considering the significant role of immune cells in inflammation-induced liver damage, it is very important to evaluate the therapeutic effects of MSCs on localized hepatic inflammatory responses. MSCs perform their immunomodulatory functions by cell-cell interaction or by producing soluble factors such as cytokines and extracellular vesicles (**Figure 4**).

#### 4.1.1 Impact on Differentiation and Function of DCs

A study by Yi Zhang et al. (109) Showed that injection of MSCs in the model of liver tissue damage induced by LPS and P. acnes (in mice) stimulates the differentiation of CD11c^+^ B220^-^ precursors to CD11c^+^, MHCII^hi^, CD80^low^, CD86^low^ tolerogenic dendritic cells. Further studies showed that MSC-derived prostaglandin E2 (PGE2) binds to the EP4 receptor on the surface of precursors of DCs and plays a key role in their differentiation into regulatory DCs in a phosphoinositide-3-kinase-dependent manner. These DCs produce more TGF-b and IL-10 than conventional DCs and stimulate the differentiation of CD4 ^+^ T cells into Treg cells (110). Therefore, these dendritic cells help improve inflammatory damage in the liver by suppressing the activity and differentiation of inflammatory T cells, including Th1 cells (109).

#### 4.1.2 Impact on Macrophages

MSCs play an essential role in modulating macrophages’ M1/M2 axis. It has been shown that the co-culture of bone marrow-derived macrophages with MSCs directs their differentiation through the Hippo-dependent pathway to M2 (111).

As mentioned, monocytes/macrophages play a very important role in liver fibrosis. In mice, Ly6C^hi^ macrophages activate HSCs by producing various cytokines and chemokines, including TGF-β, TNF-α, PDGF, MCP1, IL-1β, CCL5, and CCL3 (62). However, Ly6C^lo^ macrophages suppress the functions of HSCs and stimulate apoptosis in these cells by producing and secreting MMPs 12 and 13 and positive regulation of TNF-induced apoptosis ligand (TRAIL) (112). A study by Yuan-hui Li et al. Showed that transplantation of bone marrow-derived MSCs (BM-MSCs) in C57BL/6 J mice with liver fibrosis reduces serum alanine aminotransferase (ALT) levels and collagen deposition in the liver tissue. A sharp decrease in profibrogenic cytokines including IL-1β, TNF-α, and PDGF derived from profibrotic cells such as Ly6C^hi^ macrophages has also been observed in mice receiving MSCs (113). In fact, transplantation of BM-MSCs has prevented the activation of HSCs through an indirect mechanism by inhibiting Ly6C^hi^ macrophages’ function. These macrophages are the most common cells in the fibrotic liver, which increase in proportion to the healthy liver as the liver fibrosis. It is noteworthy that during the treatment of these mice with BM-MSCs, a phenotypic change occurs in macrophages from the Ly6C^hi^ profibrotic subtype to the regenerative Ly6C^low^, IL-4, IL-10 producing subtype. After BM-MSC transplantation in these mice, the ratio of Ly6C^hi^/Ly6C^low^ macrophages was reduced by 51% compared to the PBS-treated control group. Therefore, treatment of mouse liver fibrosis model with allogeneic BM-MSCs helps improve liver tissue by suppressing cells involved in liver fibrosis on the one hand and by expanding fibrosis inhibitory cells on the other hand (113).

#### 4.1.3 Neutrophils Migration Suppression

The results of previous studies show that in IRI-related liver injury, innate immune responses play a significant role in inflammatory responses (114). Neutrophils are the most abundant in the bloodstream and reach the site of liver damage as the first leukocytes (115). It has been reported that the severity of the disease and liver damage in I/R and alcoholic liver disease, the amount of neutrophils in the liver tissue is directly related to the severity of the injury (115, 116). However, the induction of liver damage in animals whose neutrophils decreased before induction leads to the limitation of liver damage (117). Therefore, suppressing the harmful responses of these cells can help improve the disease condition (118). The results of a study conducted in 2018 by Shihui Li et al. Show that the use of mesenchymal stem cells in rats with induced liver damage reduces liver damage by lowering neutrophil recruitment and chemotaxis compared to the control group (119).

A chemokine receptor called CXC chemokine receptor 2 (CXCR2) regulates the neutrophil release from bone marrow and its chemotaxis to the inflammation site (120). This study shows that the expression of this receptor on neutrophils in rats with liver damage is significantly reduced after the injection of MSCs (119). In addition, the expression of CXCL2, which is one of the most important chemokines in neutrophil recruitment, decreases in liver ischemic lobes compared to the control group in the experiment. Further analysis of neutrophils isolated from rats with liver damage treated with MSCs showed that p38 MAPK phosphorylation in these neutrophils was the main cause of decreased CXCR2 expression at the cell surface. Interestingly, inhibition of the MAPK p38 phosphorylating enzyme reduces the therapeutic potential of MSCs (119). In addition, since liver macrophages are the main sources of CXCL2-producing and neutrophil recruiting (94), treatment with MSCs by inhibiting NF-κB p65 phosphorylation has been shown to reduce the expression and production of CXCL2 by these cells. Therefore, in general, the use of MSCs reduces the migration of neutrophils to the liver and limits liver damage by reducing the expression of CXCR2 on the surface of neutrophils and reducing the CXCL2 expression and production by liver macrophages. As a result of MSCs therapy, the concentration of liver enzymes in plasma reduces (119). Also, a reduction in hepatocyte apoptosis, improvement in liver function, and a pathological improvement in liver tissue are seen.

#### 4.1.4 Suppression of Activation and Migration of CD4^+^ T Cells

In general, the use of MSCs to treat liver disease has been shown to reduce the activation and chemotaxis of TCD4^+^ cells (121). Examination of the expression of different markers of TCD4^+^ cells isolated from liver injury mice treated with MSCs shows that the expression of CCR7, CXCR3 and CCR5 chemokine receptors is significantly reduced. Also, the production of CXCL9, CCL3, CXCL10, and CCL21 chemokines in the mice’s damaged liver is reduced in the treatment with MSCs. In addition, CD69 and CD44 expression are reduced in MSCs treated mice, thereby suppressing TCD4 + cell activation. Serum concentrations of IFN-γ and TNF-α cytokines in these mice also indicate suppression of CD4 + T cell differentiation into Th1 cells (109, 122).

#### 4.1.5 Impact on the Th17/Treg Axis

Due to the importance of the proven role of IL-17 in liver disease (123), the study of MSCs’ effects on the IL-17 producing cells is of particular significance. As shown in previous studies, the imbalance in Treg/TH17 is associated with many liver diseases, including autoimmune hepatitis, alcoholic liver disease, and chronic hepatitis B (124).

A study by Qi-Hong Chen et al. Shows that the co-culture of MSCs with LPS-stimulated CD4 + T cell populations can induce plasticity in completely differentiated Th17 cells and convert them into functional Treg cells. The use of an anti-HGF antibody to eliminate and inhibit the effects of this factor leads to the inhibition of the modulatory effects of MSCs by regulating the Th17/Treg equilibrium (125).

In a study by Neda Milosavljevic et al., The effect of transplanted MSCs on Th17 cell responses in liver injury was investigated. IL-17 produced by Th17 cells by activating HSCs increases their production of a-SMA, type 1 collagen and TGF-b1. Intravascular injection of BM-MSCs in the calcium tetrachloride(CCL4)-induced fibrotic liver C57BL/6 mice showed a decrease in the number of IL-17 producing Th17 cells and IL-17 serum level, and also an increase in the serum level immune system suppressory factors such as IL-10, IDO and quinornine. Evaluation of liver tissue damage by H&E staining (to evaluate hepatocyte damage and lobular centre congestion with inflammatory cell infiltration) and Sirius red staining (to assess collagen deposition) in hepatic tissue of MSCs treated mice compared with CCL4-induced mice showed a significant reduction in the size of the stained area in dense fibrous tissue (126).

#### 4.1.6 Impact on the NKT17/NKTreg Axis

In addition to Th17 cells, NKT cells also play a role in the production of IL-17 involved in the pathogenesis of the liver disease (127). Intracellular staining of mononuclear cells (MNCs) isolated from damaged liver indicates that most of the IL17-producing cells in liver damage are NKT cells. A study by Neda Milosavljevic et al. showed that intravenous injection of MSCs into liver induced injuries with CCL4 and (a-GalCer) a-galactoceramide in C57BL/6 mice decreases inflammatory NKT cell function. The study results show that serum IL-17 levels are reduced in disease-induced mice treated with MSCs. Further studies show that in mice treated with MSCs compared to disease-induced mice, there was a significant reduction in IL-17-producing NKT CD49b^+^ CD31^+^ (NKT17) cells (128).

Interestingly, the application of MSCs to treat liver disease in mice treated with a-GalCer1MSC is associated with a significantly higher number of regulatory NKT FOXP3^+^ IL10^+^ (NKTreg) cells (128). Therefore, the results of this study suggest that MSCs help improve liver treatment by modulating the NKT17/NKTreg axis and reducing the harmful inflammatory responses (128). It is noteworthy that the co-culture of hepatic NKT cells *in vitro* also confirms the results obtained from the *In Vivo* studies. This function of MSCs is related to IDO and iNOS because of the presence of methyl‐DL‐tryptophan-1 (IDO inhibitor) and L‐N^G^‐monomethyl arginine citrate (iNOS inhibitor) in the cells and supernatant of injected MSCs imped the protective effect of MSCs on mice. As a result, MSC protects against acute liver damage by reducing the cytotoxicity and capacity of liver NKT17 cells to produce inflammatory cytokines in an IDO-dependent manner (128) (**Figure 3**).

### 4.2 The Role of MSCs in Liver Tissue Repair and Regeneration

Mesenchymal stem cells help repair liver tissue through various mechanisms, and there is a very complex relationship to their therapeutic function, which is discussed in more detail below.

#### 4.2.1 Reduction of Fibrosis and Impact on the Function of HSCs

MSCs co-culture with HSCs promotes their apoptosis and reduce the production of ECM components such as collagen from these cells. Reducing the synthesis of ECM components is very important in preventing fibrosis progression and can play an essential role in improving the disease (87, 129). In addition, the indirect culture of MSCs with HSC-related LX2 cell lines has been shown to reduce the proliferation of these cells by producing and secreting inflammatory factors such as IL-6, IL-8, and HGF (130). The results of recent studies show that MSCs suppress the activation of HSCs by producing TNFα-stimulated gene-6 (TSG6) and increase the expression of some stem cell markers in these cells (131). *In vitro* studies show that HSCs-derived stem cell-like cells can form organoids that help liver tissue regeneration.

#### 4.2.2 Impact on LSECs

Co-culture of MSCs with endothelial progenitor cells to investigate the effects of these cells on hepatic sinusoidal epithelial cells has been shown to increase endothelial precursor cell proliferation and angiogenic capacity by affecting PDGF and Notch-associated receptors and their downstream signaling pathways (132).

Injection of MSCs into concanavalin-A (Con-A) induced liver disease mice improves liver disease by suppressing apoptosis in LSECs and hepatocytes and lowering serum transaminase enzyme levels (133).

#### 4.2.3 Effect on Hepatocytes

The ability of MSC to differentiate into hepatocyte-like cells has been demonstrated in the *in vivo* and *in vitro* studies. This ability compensates for hepatocyte reduction through apoptosis during various liver damage. Experimental results show that MSCs can differentiate into hepatocyte-like cells (HLCs) in the presence of specific growth factors such as EGF, OSM, HGF and FGF (134, 135). Also, mimicry of the hepatic fibrosis microenvironment stimulates the differentiation of MSCs into hepatocyte-like cells by using 50 g/l of fibrotic tissue extract of rat liver at a faster rate than growth factors (136).

After differentiation, MSCs exhibit the morphology and function of hepatocytes. Analysis of differentiated cells after human Wharton’s jelly-derived MSCs (hWJ-MSCs) transplantation in rats shows the presence of cells in rat liver with the expression of human hepatocyte markers such as alpha photoprotein (AFP), CK18, CK19 and albumin. These newly differentiated cells have no sign of rat hepatocyte markers, indicating that hWJ-MSCs differentiate into hepatocyte-like cells after migration to the liver (137, 138). Also, the results of a study conducted by Jae Yeon Kim show that injection of MSCs and their supernatant into (D-galactosamine (D-gal) mediated liver damage) rats reduces hepatocytes apoptosis, and a threefold increase occurs in their proliferation. MSCs appear to increase hepatocyte proliferation by activating the IL-6 signaling pathway mediated by rno-miR-21-5p (139).

Therefore, in general, it can be said that the use of MSCs induces the repair and regeneration of liver tissue through immune responses modulation, differentiation into HLCs, increased proliferation and decreased apoptosis in hepatocytes, increased apoptosis and reduced function of HSCs and improve the function of LSECs.

## 5 Clinical Applications of MSCs for the Treatment of Liver Disease

### 5.1 Cell Source, Injection Method and Injection Dose

According to our search in the National Institutes of Health (NIH), there are currently 61 active clinical trials using MSCs to treat various liver diseases, including Cirrhosis, fibrosis, acute-on-chronic Liver Failure, and hepatitis viruses related liver failure. **Table 3** summarizes some of these studies. MSCs in these studies were isolated from various tissues, including umbilical cord (UC), bone marrow (BM) and adipose tissue (AD). According to the results of multiple studies on the function of MSCs derived from different tissues, UC-MSCs are used in clinical applications more than others due to features such as (1) providing significant amounts of MSCs compared to BM (2) non-invasive isolation method (3) higher self-renewability and differentiation capacity of UC-MSCs in comparison with BM-MSCs and (4) lower immunogenicity (140, 141).

**Table 3:** MSCs based clinical trails.

Liver diseases	Intervention Model	Estimated Enrollment	Source of MSCs	Route	Phase	Dose	Date	NTC number
Acute-on-Chronic Liver Failure	Parallel Assignment	45	N/A	Intravenous (peripheral vein)	Phase 1 and Phase 2	once a week for 4 weeks, 1-10 × 10^5^ cell/kg	2019	NCT03863002
Acute-on-chronic Liver Failure	Parallel Assignment	200	N/A	Intravenous (peripheral vein)	N/A	3 times at week 0, 4 and 8, 1 × 10^6^ cell/kg	2018	NCT03668171
Alcoholic Liver Cirrhosis	Single Group Assignment	10	Bone Marrow	Hepatic Artery injection	Phase 1	A single dose of 4.5-5.5 × 10^7^ cell	2019	NCT03838250
Decompensate Cirrhotic Patients With Pioglitazone	Single Group Assignment	3	Bone Marrow	Intravenous (portal vein)	Phase 1	2 doses at with week intervals	2014	NCT01454336
Decompensated Alcoholic Cirrhosis	Sequential Assignment	36	Umbilical Cord	Intravenous	Phase 1	0.5-2 × 10^6^ cell/kg	2021	NCT05155657
Decompensated Hepatitis B Cirrhosis	Single Group Assignment	30	Umbilical Cord	Intravenous	N/A	2 doses with 24 week intervals, 1 × 10^8^ cell	2021	NCT05106972
Decompensated liver Cirrhosis	Parallel Assignment	240	Umbilical Cord	Intravenous	Phase 2	3 doses at week 0, week 4, week 8, 6 × 10^7^ cell/kg	2021	NCT05121870
Decompensated Liver Cirrhosis	Parallel Assignment	45	Umbilical Cord	Intravenous	Phase 1 and Phase 2	3 doses with 4 week intervals, 0.5 × 10^6^ cell/kg	2011	NCT01342250
End-stage Liver Disease (Cirrhosis)	Single Group Assignment	30	N/A	Intravenous	Phase 1 and Phase 2	N/A	2018	NCT03460795
HBV-Related Acute-on-Chronic Liver Failure	Parallel Assignment	261	Umbilical Cord Blood	Intravenous (peripheral vein)	Phase 2	1.once a week for 4 weeks once a week for 8 weeks	2016	NCT02812121
HBV-related Liver Cirrhosis	Parallel Assignment	240	Umbilical cord	Intravenous	Phase 1 and Phase 2	1 × 10^6^ cell/kg	2012	NCT01728727
Hepatitis B mediated Liver Cirrhosis	Single Group Assignment	12	Umbilical Cord	Intravenous	Phase 1 and Phase 2	A single dose of 1 × 10^8^ cell	2020	NCT04357600
Liver Cirrhosis	Single Group Assignment	30	Bone marrow	Intravenous (peripheral or the portal vein)	Phase 1 and Phase 2	3-4 × 10^7^	2007	NCT00420134
Liver Cirrhosis	Single Group Assignment	50	Menstrual Blood	Intravenous	Phase 1 and Phase 2	4 time in 2 week 1 × 10^6^ cell/kg	2012	NCT01483248
Liver Cirrhosis	Parallel Assignment	200	Umbilical Cord	Intravenous	Phase 2	3 doses with 3 week intervals, 1 × 10^6^ cell/kg	2019	NCT03945487
Liver Cirrhosis	Single Group Assignment	20	bone marrow	Intravenous	Phase 1 and Phase 2	A single dose of 0.5 - 1×10^6^ cell/kg	2018	NCT03626090
Liver Cirrhosis	Parallel Assignment	266	Umbilical Cord	Intravenous	Phase 1 and Phase 2	3 doses with 4 week intervals, 0.5 × 10^6^ cell/kg	2018	NCT01220492
Liver Cirrhosis	Single Group Assignment	4	Adipose tissue	Intrahepatic Arterial Administration	N/A	N/A	2010	NCT01062750

HBV, Hepatitis B virus; MSCs, Mesenchymal stromal/stem cells; EVs, Extracellular vesicles; TGF-β, Transforming growth factor-beta; NF-kB, Nuclear factor kappa-B; MMPs, Matrix metalloproteinase; NLRP, Nucleotide-binding oligomerization domain; ALT, alanine aminotransferase; AST, Aspartate transaminase; ALP, Alkaline Phosphatase; BM, Bone marrow; UC, umbilical cord; AD, adipose tissue; N/A, Not Applicable.

Injection methods such as intravenous (portal vein or peripheral), intrahepatic artery and intrasplenic injection are used in the transplantation of MSCs. The intravenous (IV) peripheral injection is more used in studies due to ease of identification and less invasive method. However, animal studies have shown that approximately 60% of IV-injected cells never reach the liver and accumulate in tissues like the lungs and kidneys. Due to the existence of different injection methods, in a study conducted by MEM Amer et al., It was shown that injection of MSCs through the portal vein shows better therapeutic results in patients compared to intrasplenic injection (142). As shown in **Table 3**, the number of injections doses, the time interval between injections, and the number of injection cells in the studies vary depending on the different groups’ set-up protocols.

### 5.2 Mechanisms Involved in Liver Disease Treatment After MSCs Transplantation

A phase 1 clinical trial conducted by Kharaziha et al. Showed that the use of BM-MSCs improved patients’ symptoms of uncompensated Cirrhosis. Various scores related to liver healing indicate improvement of the disease without side effects (143). Also, the results of phase 2 of this study, which was performed on patients with alcoholic cirrhosis, showed a reduction in tissue fibrosis and improved liver histology in patients after mesenchymal stem cell transplantation through the hepatic artery (143, 144). Due to the importance of safety in cell therapy studies, a study conducted by Alimoghaddam K et al. has examined this issue in the transplantation of MSCs in various liver diseases, which shows the improvement of liver function in patients without any side effects (145).

A study by Ani Sun et al. Showed that patients receiving routine treatment (RT) in combination with autologous BM-MSCs transplantation had better clinical symptoms such as decreased fatigue and ascites, increased appetite, and abdominal distension than patients receiving only routine treatment. This study also showed that liver function significantly improved in patients receiving combination therapy in terms of MELD (model for end-stage liver disease) and Child-Pugh scores, serum albumin, total bilirubin, aspartate aminotransferase(AST), alanine aminotransferase(ALT), and coagulation function (146).

As mentioned in the *in vitro* studies and animal models of liver disease, the application of MSCs helps improve liver function with different mechanisms in humans. A study conducted by Lanman Xu in 2014 showed that BM-MSCs improve the symptoms of patients with HBV associated Cirrhosis. The findings in this study showed that the number and ratio of Treg cells in PBMCs isolated from patients increased; however, the number of Th17 cells in patients treated with BM-MSCs decreased. Also, extraction of total RNA and evaluation of the expression of Foxp3 and RORγt transcription factors by Real-time polymerase chain reaction (RT-PCR) showed an increase in Foxp3 expression and a decrease in RORγt expression compared to the control group. Also, examination of patients’ serum in the first week after transplantation showed an increase in TGF-B and a decrease in inflammatory cytokines such as IL1-B, IL-6 and TNF-a. Therefore, in general, transplantation of MSCs by modulating the Treg/Th17 axis and modulating the production of inflammatory cytokines helps improve liver inflammation and patient conditions (147).

The results of a study conducted in 2020 by Federica Casiraghi et al. Show that intravenous injection of MSCs before liver transplantation does not improve the parameters of chance tissue compared to the group that received this treatment. However, a one-year follow-up of patients shows a slight increase in the circulating Treg/memory Treg and tolerant NK subset (CD56^bright^ NK cells) over baseline (not significant) in MSC-treated compared to the control group (148).

As shown in **Table 2**, the disadvantages of using MSCs, encourage researchers to find safer and more efficient methods. As has been proven in many studies, MSCs perform their therapeutic functions after transplantation in two ways: cell-to-cell communication and secretion of soluble factors (paracrine effect). EVs are one of the main factors in this type (149).

## 6 MSCs-Derived EVs Application in Liver Disease Treatment

EVs are a significant component of intercellular communication that affect the actions of the target cell by transporting various substances, including proteins, lipids, and nucleic acids, from the producing cell to the target cell (150). These vesicles are divided into microvesicles (MVs), apoptotic bodies and exosomes according to their size, internal contents, biogenesis and different surface markers (151). However, the fourth group of these vesicles, smaller than the rest, has been identified as named exomer (152). Each of these vesicles has different contents, typically containing various proteins (including enzymes, cytokines, growth factors and heat shock proteins), different types of nucleic acids (including DNA, microRNA and lncRNAs) and different types of lipids (153). These molecules change target cell function after transferring by EVs bilipid membrane. As a consequence, and depending on the cell type and its physiological conditions, exosomes can lead to differentiation, increase proliferation and survival, or lead to increased apoptosis and decreased cell activity (154).

Many studies have examined the therapeutic effects of MSC derived EVs (MSC-EVs) in different diseases such as pulmonary fibrosis, osteoporosis, skin diseases, cardiovascular diseases, and various liver-related diseases. The results of new studies show that the application of MSCs-EVs has similar therapeutic effects to MSCs (155–157). In fact, the use of EVs is cell-free cell therapy because it preserves the benefits associated with cell therapy and bypasses its disadvantages. These benefits include: (1) lack of immunogenicity, (2) the ability to store them easily, (3) drug loading and increase their efficiency as a drug delivery system and (4) application as a ready-to-use drug (cryopreservation of EVs) (158).

Given that the results of many *in vitro* and animal model studies have shown that MSCs can migrate to TME, it is thought to be they can be used as carriers for tumor-targeted therapies (159). However, there is little clinical evidence of MSCs recruitment in hepatocellular carcinoma (160). Also, due to the proliferative and differentiating characteristics of MSCs, considering the possibility of malignant transformation and promotion of tumor progression by these cells, most studies are still in the preclinical stage (159). Therefore, to use the therapeutic properties of these cells, researchers used MSC-EVs that do not have these deficiencies (161). Recent studies show that adipose tissue MSCs derived exosomes (MSC-Ex) can increase the chemical sensitivity of liver cancer cells by affecting the mTOR related signaling pathway and leading to increased expression of chemo-sensitive related genes in cancer cells (162). Further studies have shown that these exosomes perform this function by transferring miR-199a to cancer cells (162). MiR-222-3p in BM-MSC-Ex has also been shown to inhibit cancer cell proliferation and increase their apoptosis (163). Another study showed that miR-302a, carried by UC-MSC-Ex, suppressed cyclin D1 as well as the AKT signaling pathway and thereby suppress tumor progression (164). Also, various anti-tumour drugs can be loaded into exosomes and used as targeted therapy (165). In addition, it has been shown that exosomes can be engineered to migrate to the target site, integrate with cancer cells, and deliver anti-tumor drugs to them (166, 167). However, the application of MSC-Ex is still in its initiation, and further studies are needed for their optimal use.

As mentioned, due to the size of MSCs, when injected intravenously, they accumulate in the lungs while EVs are much smaller and can migrate to the site of injury (168). MSC-EVs exert their therapeutic functions through 3 mechanisms that have been studied *in vitro*, pre-clinical, and clinical trials. These mechanisms include (1) proliferation induction/apoptosis suppression, (2) modulation of immune system responses, (3) reduction of fibrosis. **Table 4** summarizes several new studies on the application of MSC-EVs (**Figure 4**).

**Table 4:** Example of studies in the field of MSC-EV application in experimental models of liver injury and their therapeutic mechanisms.

Injury model	Source of MSCs	Acute Or chronic phase	EVs type	Route of administration	Dosage (vesicles/animal)	Effect(s)	Mechanism(s)	Year	Ref.
CCl4-induced acute liver injury(mouse)	hUC-MSCs	Chronic	Exosome	Intrahepatic	250 µg	Inhibited hepatocyte apoptosis Reduce liver fibrosis Reduce the serum levels of HA	Suppressed TGF-b signaling and inhibited EMT Reduced collagen-1 and 3 expression	2013	(169)
Hepatic ischemia-reperfusion (mouse)	mBM-MSC	Acute	EVs	Intravenous	2 × 10^10^	Reduction of inflammatory mediators inhibition of Apoptosis Increase the number of F4/80 positive cells	Suppress NF-κB activity increased CXCL1 release from AML12 hepatocytes *in vitro*	2017	(170)
*In vitro* ischemia/reperfusion Partial hepatectomy (mouse)	mAD-MSC	Acute	Secretome (EVs + other soluble factors)	Intravenous	N/A	Reduce serum IL-6 and TNF-a levels Reduce serum transaminases Accelerate liver regeneration Increase the hepatocyte proliferation	Increased p-STAT3 and PCNA expression Decreased hepatic expression of SOCS3 increased SIRT1 Increase in survival genes (e.g., Bcl-xL and Mcl-1)	2017	(171)
Thioacetamide induced (rat)	Human embryonic MSC	Chronic	EVs	Intrahepatic	350 µg	Reduction of fibrosis Reduction inflammation	upregulation in MMP9 and MMP13 upregulation of BCL-2 upregulation of TGF-β1 and IL-10 downregulation of Col1α, αSMA and TIMP1 downregulation BAX, TNFα and IL-2	2018	(172)
CCl4-induced acute liver injury(mouse)	hUC-MSCs	Acute	Exosome	Intravenous Or intragastric	8, 16, and 32 mg/kg	Reduction of oxidative stress inhibition of Apoptosis Increased cell viability	Reduced levels of ROS Upregulated Bcl2 expression	2017	(173)
S.japonicum-infected mice	hUC-MSCs	Chronic	EVs	intravenous	3 × 10^9^	Reduce liver fibrosis suppress HSCs function Reduction inflammation	Reduced collagen-1 and 3 expression Reduced α-SMA expression significantly decrease TNF-α and IL-1β expression	2020	(174)

MSCs, Mesenchymal stromal/stem cells; EVs, Extracellular vesicles; TGF-β, Transforming growth factor-beta; NF-kB, Nuclear factor kappa-B; MMPs, Matrix metalloproteinase; NLRP, Nucleotide-binding oligomerization domain; ALT, alanine aminotransferase; AST, Aspartate transaminase; ALP, Alkaline Phosphatase; BM, Bone marrow; UC, umbilical cord; AD, adipose tissue; N/A, Not Applicable.

### 6.1 Stimulation of Proliferation/Suppression of Apoptosis in Hepatocytes

Examination of the effects of MSCs-derived exosomes *in vivo* shows that these vesicles suppress acetaminophen and H2O2-induced apoptosis in hepatocytes by positively regulating Bcl-xl expression (175). Also, the study shows that MSC-Ex stimulates hepatocytes proliferation in CCL4-induced liver damage in mice. Further studies show that the exosomes positively regulate the priming phase genes, which is subsequently increases the expression of Proliferating cell nuclear antigen (PCNA) and cyclin D1 in the treated group compared to the control group (175).

The results of new studies show that the use of BM-MSCs-Ex reduces apoptosis by increasing autophagy in hepatocytes. A study by Shuxian Zhao et al. Shows that the use of BM-MSCs-Ex reduces D-GaIN/LPS-induced apoptosis in rats hepatocytes. Further studies show that autophagy-related markers such as LC3 and Beclin-1 are increased and have led to autophagosomes formation by hepatocytes. Also, the expression level of apoptosis-related proteins such as Bax and cleaved caspase 3 was decreased, and the expression level of Bcl-2 (anti-apoptotic protein) was increased. Because the use of 3-Methyladenine (3-MA), an autophagy inhibitor, limited the therapeutic effects of BM-MSCs-Ex, this study attributed the main therapeutic mechanism of these vesicles to the regulation of apoptosis in an autophagy-dependent manner (176).

A study conducted by Yinpeng Jin et al. In 2018 shows that the use of AD-MSCs-EVs in the model of acute liver failure in rats increases the survival rate by more than 70% compared to the control group. Liver sequencing of rats treated with AD-MSCs-EVs demonstrated an increase in a non-coding long-stranded RNA (lncRNA) called lncRNA H19 (H19), and when the coding sequence of H19 in AD-MSCs of EVs source was knocked off, survival rates could be 40% higher than the control group. In fact, H19 encoding gene silencing reduces the survival of rats compared to the AD-MSCs-EVs treated group. The results of hepatocytes Co-culture with normal AD-MSCs-EVs and manipulated AD-MSCs-EVs (lacking H19) indicate that this lncRNA increases the proliferation of hepatocytes by the HGF/c-Met signaling pathway as well as reducing apoptosis (177).

### 6.2 Immunomodulatory Properties

The results of a study by H Haga et al. show that administration of BM-MSC-derived EVs to mice with liver injury induced by Intraperitoneal (IP) injection of TNF-a and D-gal increases the migration of protective macrophages to damaged liver tissue. Another study showed that injections of MSC-EVs into a mouse model of ischemic-reperfusion injury helped reduce inflammatory responses by modulating the expression of NLRP12 and CXCL1. This study also showed that the frequency of F4/80^+^ cells (expressed in protective macrophages) in the damaged liver increased significantly after MSCs-EVs injection (178).

Con-A-induced liver injury studies in C57/B6 male mice show extensive modulation of the immune system by AD-MSCs-Ex in the treatment group compared to the control group. The analyses showed that the expression of IL-2 (a pro-inflammatory cytokine) was associated with a significant decrease. On the other hand, the expression of cytokine TGF-β and HGF was upregulated. Also, the percentage of Treg cells among non-parenchymal liver cells (NPCs) in the three doses of AD-MSCs-Ex treated group increased significantly. Treg has been reported to be required to induce immune tolerance in this liver injury model (179). This study also compared the therapeutic effects of AD-MSC injection and one-dose injection of AD-MSCs-Ex with 3-dose injection. The present results of the study show a significant difference in the therapeutic application 3-dose injection of AD-MSCs-Ex with other groups. Histological and serum analysis also confirmed a decrease in fibrotic and necrotic areas of the liver and a decrease in ALT levels in the treated groups (177).

Using the protein chip method by Xiaoli Rong et al. showed that in treatment of D-GaIN/LPS induced damages in rats with AD-MSCs-EVs, the expression of inflammatory cytokines including IL-6, IL-1α, IL-1β, IL-1ra and IL-17 in was significantly lower than in the Phosphate-buffered saline (PBS) treatment group. Also, the level of inflammatory chemokines such as CXCL7, CXCL9, CXCL10, CCL20, CX3CL1, CINC-3, CINC-2α/β, CINC-1, CNTF, and LECAM-1 decreases in the AD-MSCs-Ev treated group compared to the control group PBS treated group. Simultaneous reduction of inflammatory cytokines and chemokines ultimately leads to a reduction in liver tissue necrosis and an increase in the survival rate of rats (180). Evaluation of inflammatory cytokines including TNF-α, IL-1, IL-2, IL-6, IL-8, and IL-10 and in liver tissue using RT-PCR showed a significant reduction in rats treated by hBM-MSCs and hBM-MSCs-Ex. However, the results of this study showed that the expression of IL-6 and IL-1 in the hBM-MSCs-Ex treatment group was significantly lower than in the hBM-MSCs treated group (181).

Also, the study of malondialdehyde (MDA) levels, which is the end product of membrane lipid peroxidation, can be used as a marker for oxidative stress and liver cell damage. Analysis in hBM-MSCs-Ex treated rats (CCL4-induced liver injury) showed a significant reduction in MDA level (181).

### 6.3 Reduce Hepatic Fibrosis

The results of a study conducted in 2019 (181) show that the administration of hBM-MSCs-Ex to rats with CCL4-induced liver damage effectively reduces liver fibrosis. Visual examination showed that the liver of mice treated with hBM-MSCs-Ex had a smooth, uniform surface and softer tissue than the control group. Sirus red and H&E staining show reduction of fibrous areas and collagen deposition in the treated liver. In addition, comparing the therapeutic effects of hBM-MSCs-Exo and hBM-MSCs indicates the better therapeutic outcome in the application of hBM-MSCs-Ex due to the collagen deposition level and Ishak fibrosis score. Examination of the hydroxyproline levels, unnecessary amino acid and a major component of collagen also shows a decrease in this amino acid in the treated group and indicates a reduction in collagen deposition. In this study, the biochemical analysis showed that the level of AST, ALT, ALP, total bilirubin (TBIL) and gamma-glutamyl transferase (γ-GT) decreases, and serum total protein (TP) levels increases in serum. Examination of Wnt/β-catenin signaling related factors including PPARγ, Wnt10b, Wnt3a, β-catenin, and WISP1 in both *in vivo* and *in vitro* conditions indicates the use of hBM- MSCs and hBM-MSCs-Ex (have better therapeutic results) reduce the expression of these factors in HSCs (*in vitro*) and with rat fibrous liver. Due to the reduction of collagen deposition and fibrotic tissue in the damaged liver, the results of this study suggest using hBM-MSCs-Ex leads to decreased HSCs function and reduced fibrosis by inhibiting the Wnt/β-catenin signaling pathway (181).

## 7 Conclusions and Future Prospects

Due to the urgent need to treat tissue degenerative diseases, methods that can accelerate tissue repair are of great value. Using MSCs as multipotent stem cells is one of these widely used methods due to its properties. As mentioned, these cells uses different mechanisms such as differentiation into hepatocyte-like cells, reducing apoptosis and increasing hepatocyte proliferation, reducing inflammation, suppressing tissue-damaging immune cells, producing various growth factors, suppressing the function of HSC, and improving the function of LSECs to improve liver diseases. The use of these cells not only prevents further damage to the liver tissue but also accelerates and increases the repair of liver tissue. MSCs migrate to the site of liver injury after intravenous injection due to their chemokine receptors and perform their therapeutic actions there. As mentioned earlier, due to the limitations of cell therapy, MSC-EVs are used today. Many studies have been conducted on the application of MSC-EVs in preclinical studies. The results of these studies indicate the potential ability of these vesicles to improve liver cell function and modulate the immune system (182). Some studies comparing the therapeutic effect of MSCs and MSC-EVs have shown that MSC-EVs have a higher therapeutic potential and better outcomes. Although the therapeutic effects of EVs on liver repair in this study are divided into three distinct mechanisms, these processes are interrelated and complement each other’s functions. In fact, the therapeutic potential of MSC-EVs likely lies in their ability to act simultaneously through multiple signaling pathways. Therefore, using these vesicles does not have cell therapy disadvantages. Due to its benefits, it can perform better therapeutic performance in liver injury animals models.

Although there are numerous clinical trials on the use of MSCs in the treatment of liver disease, to date, no clinical trials have been performed on the use of MSC-EVs despite their benefits. However, it has been used to treat many disorders, including orthopaedic disease (151), neurodegenerative disease (183), myocardial disease (184), renal disease, graft versus host disease (GvHD), pancreatic cancer and type 1 diabetes and has shown promising results (185). Despite the therapeutic potential of MSC-EVs and their beneficial effects in preclinical studies, several issues related to the clinical use of these vesicles remain unresolved. Exosomes as an EV circulate in the blood and transfer their cargos to the target cell through phagocytosis, receptor-mediated endocytosis, fusion, micropinocytosis (186). In the body, the half-life of EVs is estimated at a few minutes and are removed from the bloodstream within a few hours. Therefore, we need multiple injections to treat diseases. Due to this issue, producing EVs in an industrial scale and repeatable method is significant. It is also important to note that despite effective and proven results in animal models, the improvement of these models is usually incomplete. Therefore, more research is needed to investigate the long-term effects of MSC-EVs.

## Author Contributions

AH: Conceptualization, Investigation, Writing - original draft, Writing - review and editing, Validation. KM: Writing - review and editing, Validation. SS: Edited final version of the manuscript. SH: Supervision and final approval of the manuscript. All authors contributed to the article and approved the submitted version.

## Conflict of Interest

The authors declare that the research was conducted in the absence of any commercial or financial relationships that could be construed as a potential conflict of interest.

## Publisher’s Note

All claims expressed in this article are solely those of the authors and do not necessarily represent those of their affiliated organizations, or those of the publisher, the editors and the reviewers. Any product that may be evaluated in this article, or claim that may be made by its manufacturer, is not guaranteed or endorsed by the publisher.

## Abbreviations

AD, adipose tissue; AFP, alpha photoprotein; ALF, Acute liver failure; ALP, Alkaline Phosphatase; ALT, alanine aminotransferase; AST, Aspartate transaminase; BM, Bone marrow; CMV, Cytomegalovirus; Con-A, concanavalin-A; DCs, Dendritic cells; ECM, extracellular matrix; EVs, Extracellular vesicles; FLD, Fatty liver disease; HGF, hepatocyte growth factor; HLCs, hepatocyte-like cells; HMGB1, High mobility group box 1; HSCs, hepatic stellate cells; IDO, indolamine 2,3-dioxygenase; IV, intravenous(; KCs, Kupffer cells; KLF2, Krüppel factor 2 transcription factor; LDs, Liver disease; LPS, Lipopolysaccharide; LSECs, hepatic sinusoidal endothelial cells; MMPs, Matrix metalloproteinase; MSCs, Mesenchymal stromal/stem cells; NF-kB, Nuclear factor kappa-B; NK, Natural killer cell; NLRP, Nucleotide-binding oligomerization domain; ROS, Reactive oxygen species; TGF-β, Transforming growth factor-beta; TLR, Toll-like receptor; TSG6, TNFα-stimulated gene-6; Tumor necrosis factor, TNF; TXA2, Thromboxane A2; UC, umbilical cord; WJ, Wharton’s jelly.
